# Skin phototypes, UV exposure, and socioeconomic vulnerability in a region of the Brazilian Amazon

**DOI:** 10.1007/s00484-025-03059-3

**Published:** 2026-02-24

**Authors:** Pericles Alves, Vandoir Bourscheidt, Gustavo Ávila Maquiné, Regiane Luiza da Costa, Vanessa Colares Magalhães Alves, Paula Regina Humbelino de Melo, Maria Adriana Moreira

**Affiliations:** 1 Department of Environmental Sciences (DCAm), Center for Biological and Health Sciences (CCBS), Graduate Program in Environmental Sciences (PPGCAm), Federal University of São Carlos (UFSCar), São Carlos, São Paulo Brazil; 2 Assistant Professor II, Institute of Education, Agriculture, and Environment (IEAA), Federal University of Amazonas (UFAM), Humaitá, Amazonas 69800-000 Brazil; 3 Associate Professor, Department of Environmental Sciences (DCAm), Center for Biological and Health Sciences (CCBS), Graduate Program in Environmental Sciences (PPGCAm), Federal University of São Carlos (UFSCar), São Carlos, São Paulo 13565-905 Brazil; 4 Dermatologist at the Alfredo da Matta Hospital Foundation, Manaus, Brazil; 5 Ph.D. in Environmental Sciences from the Environmental Sciences Program at the Federal University of São Carlos, São Carlos, São Paulo 13565-905 Brazil; 6 Physiotherapist, Coordinator of Permanent Health Education at the Municipal Health Secretary of Manicoré, Manicoré, Amazonas 69800-000 Brazil; 7 Adjunct Professor II, Institute of Education, Agriculture, and Environment (IEAA), Federal University of Amazonas (UFAM), Humaitá, Amazonas 69800-000 Brazil; 8 Nurse, Municipal Health Secretary of Manicoré, Amazonas, and Master’s student in the Professional Program in Family Health (PROFSAÚDE) at FIOCRUZ, Manicoré, Brazil

**Keywords:** Fitzpatrick scale, UV radiation, Photoprotection, Skin cancer, Southern amazonas

## Abstract

This study aimed to describe the skin phototypes of the sampled population in a region of the Brazilian Amazon using the Fitzpatrick scale, as well as to investigate sun exposure habits and associate photoprotection practices with socioeconomic vulnerability conditions. Data were obtained from a household survey conducted in 2022 by Community Health Agents of three Municipal Health Departments, covering 1,442 urban adults. Descriptive statistics, Pearson’s Chi-squared test with Yates’ correction, and Odds Ratios were applied to assess associations between socioeconomic vulnerability and photoprotection practices. Results showed a predominance of melano-protected phototypes (V and VI, 49.2%), followed by melano-competent (III and IV, 43.9%) and melano-compromised (I and II, 7%). While lighter phototypes often adopt sun protection, darker phototypes face greater exposure linked to work and culture. Sunscreen adherence was low across all phototypes, with alternative methods, such as wearing hats and clothing, being more common. Socioeconomic vulnerability increased the likelihood of not adopting photoprotection practices by 2.28 times. These findings highlight that low sunscreen adherence, combined with socioeconomic vulnerability, substantially increases the risk of inadequate photoprotection in the Brazilian Amazon.

## Introduction

The Global Cancer Observatory, through the International Agency for Research on Cancer (IARC), estimated approximately 1.2 million new cases of non-melanoma skin cancer (NMSC) worldwide in 2022 (Bray, et al., 2024). In Brazil, according to estimates from the National Cancer Institute (INCA) for the 2020–2022 period, NMSC was the most common type of cancer, with approximately 177,000 new cases expected annually, representing more than 27% of all malignant tumors in the human body (INCA, 2019). For the 2023–2025 period, INCA projects 221,000 new cases, accounting for 31.3% of all other cancer cases (INCA, 2022).

This high burden of NMSC highlights the importance of understanding its main etiological factor. In this context, solar radiation, particularly ultraviolet (UV) radiation, plays a critical role as the primary driver of skin cancer. Health research highlights that UV radiation impacts human health in both harmful (Saucedo et al. 2020) and beneficial ways (Juzeniene and Moan 2012). Cumulative exposure to UV radiation over a lifetime is especially relevant, as it increases the risk of skin damage, including premature aging and skin cancer. In addition to environmental and behavioral factors, genetic aspects, such as skin phototype, influence sensitivity to UV radiation (Al-Sadek and Yusuf 2024; Juzeniene, et al., 2011).

Given the critical role of UV radiation in skin cancer development, the Fitzpatrick scale emerges as a key tool to classify skin phototypes. With six phototypes (Fitzpatrick 1988), it serves as a reference for phototherapy protocols (Young et al. 2017; Diffey et al. 1997) and skin cancer prevention campaigns, such as “Dezembro Laranja” (SBD 2021a). In the Amazon, where solar exposure is intense and resources are limited, tools such as the Fitzpatrick scale are essential for understanding the population’s susceptibility to UV radiation. However, this classification is subjective, as skin sensitivity is influenced by individual factors such as exposure habits (IARC 2012), genetic predispositions (Al-Sadek and Yusuf 2024), and health conditions (Kennedy, et al., 2021; Nehal and Bichakjian 2018; Hussein 2005). In our study, this limitation implies that phototype classification may not fully capture the interindividual skin variability observed in a highly miscegenated population such as the Brazilian Amazon.

In the Amazon region, continuous ground-based UV measurements are extremely scarce, with only two publications reporting data: one for the city of Humaitá in Amazonas (Alves, et al., 2022) and another for Santarém in Pará (Reis, et al., 2022). This limitation restricts public health specialists’ ability to effectively guide the population on safe sun exposure times and necessary precautions for different skin types. Many of the populations inhabiting the region rely heavily on outdoor activities, such as agriculture and fishing, for their livelihoods. These activities expose individuals to prolonged periods of sun exposure, significantly increasing the risk of skin damage and other health issues associated with solar radiation (Malinović-Milićević, et al., 2022; Silva et al. 2020; Modenese et al. 2018; Franco et al. 2016; Dantas, et al., 2016).

In this context, recent studies, such as Alves et al. (2025), highlight the urgency of continuous monitoring UV radiation in the region. Indeed, the authors identified significant upward trends in irradiances at wavelengths of 324 and 380 nm in the southern mesoregion of Amazonas over an 18-year period. The authors attribute this behavior to a notable decrease in cloud optical thickness (Jebar et al. 2020), which is strongly correlated with changes in land cover and use (Stabile, et al., 2020; Wang, et al., 2016). These changes are driven by agricultural and logging developments in the region (Moura et al. 2019; Pontes et al. 2016).

The scarcity of continuous UV monitoring in the Brazilian Amazon therefore represents a critical knowledge gap. Addressing this gap was one of the main motivations for conducting the present study, which aims to provide evidence to better understand sun exposure risks and guide public health strategies in the region.

This lack of data is particularly concerning when combined with the socioeconomic vulnerability of the populations inhabiting the region. Additionally, beyond environmental concerns, these populations face significant socioeconomic challenges, including limited access to healthcare, education, and basic infrastructure (Garnelo et al. 2020; Mesquita, et al., 2020). Such challenges exacerbate the socio-environmental issues affecting these communities, reducing awareness of the risks associated with sun exposure and limiting access to appropriate photoprotection measures to mitigate the impacts of prolonged UV radiation exposure (Costa, et al., 2022; Mesquita, et al., 2020).

Therefore, this study aimed to describe the skin phototypes of the sampled population in a region of the Brazilian Amazon using the Fitzpatrick scale, as well as to investigate sun exposure habits and associate photoprotection practices with socioeconomic vulnerability conditions.

## Materials and methods

### Study area description

**Fig. 1:**
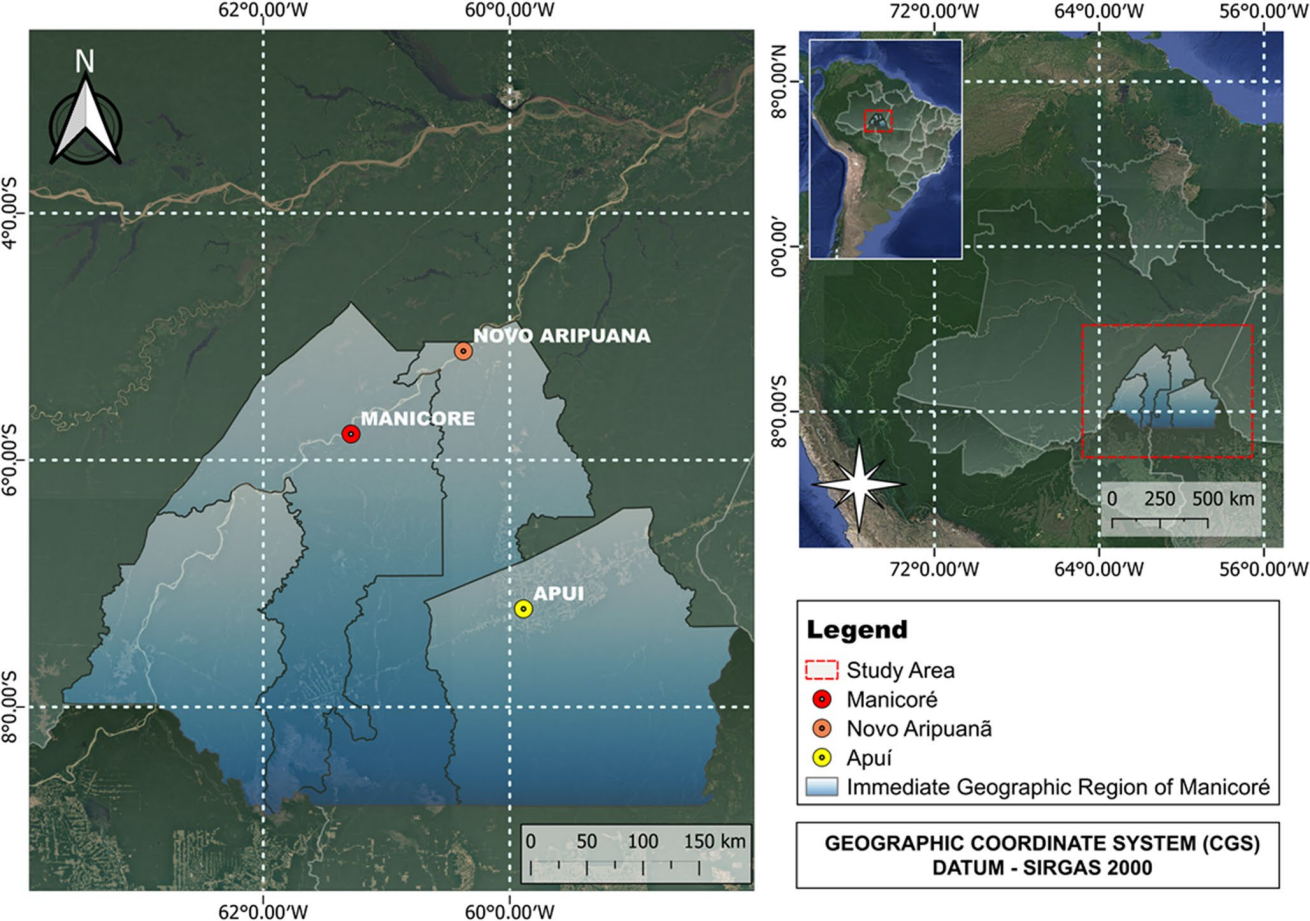
Location of the study area, highlighting the municipalities of the Immediate Geographic Region of Manicoré, where data collection was conducted

This research was conducted in the Immediate Geographic Region of Manicoré (RGIM), which is part of the Intermediate Region of Lábrea (RIL) in Amazonas, Brazil (IBGE 2017). Data collection focused on the municipalities of Manicoré (48,300 km²), Novo Aripuanã (41,200 km²), and Apuí (54,200 km²) (Fig. 1). According to the 2022 Demographic Census by the Brazilian Institute of Geography and Statistics (IBGE), these municipalities have populations of approximately 54,000 in Manicoré (1.12 inhabitants/km²), 24,000 in Novo Aripuanã (0.58 inhabitants/km²), and 21,000 in Apuí (0.38 inhabitants/km²) (IBGE 2022).

These municipalities share common characteristics associated with their location in the Amazon, such as a tropical monsoon climate (Am) marked by three distinct periods: rainy (October to April), dry (June to August), and transitional (May and September) (Silva Martins, et al., 2023; Souza, et al., 2022). The predominant vegetation is tropical rainforest, with vast biodiversity, while the local economy relies primarily on subsistence agriculture, fishing, vegetal and mineral extractivism, and livestock farming (Limberger and Silva 2016). The average monthly wage of formal workers ranges from 1.6 to 2.0 minimum wages, with only 7.1%, 8.8%, and 7.3% of the population in Manicoré, Novo Aripuanã, and Apuí, respectively, holding formal employment (IBGE 2022). These municipalities are also traversed by significant rivers, such as the Madeira River, which is essential for transportation and the flow of goods due to limited road access (Tavares and Cordeiro 2017). Populations in these areas face challenges related to geographic isolation, with infrastructure and public services often restricted, especially in riverside communities (Garnelo et al. 2020).

### Data collection and processing

Information on phototypes, sun exposure habits, photoprotection practices, and individuals in situations of socioeconomic vulnerability was obtained from the databases of the Municipal Health Departments (SEMSAs) of Manicoré, Novo Aripuanã, and Apuí. These data are based on the year 2022.

In that year, SEMSAs, through their Community Health Agents (ACS), conducted a household survey covering approximately 3% of the urban populations (based on the 2010 IBGE Census). Only individuals aged 18 and older were interviewed. Rural populations were not included due to the significant logistical challenges in reaching remote areas, which demand extensive human resources, infrastructure, and transportation. Consequently, the total sample population consisted of 1,442 respondents within the Immediate Geographic Region of Manicoré.

### Data presentation and statistical analysis

The percentage distributions of populations describing phototypes, sun exposure habits, photoprotection practices, and individuals in situations of socioeconomic vulnerability are presented using bar charts. Additionally, a 3D histogram was created to illustrate the behavior of population frequency percentages in terms of household gross income and sun exposure time. For the proportions of phototypes, 95% confidence intervals (CIs) were calculated using the Wilson method for binomial data, based on the total sample size.

To investigate the association between socioeconomic vulnerability and photoprotection practices, a 2 × 2 contingency table was constructed, and Pearson’s Chi-squared test with Yates’ continuity correction was applied, using a 95% significance level (Serra et al. 2019; Haber 1980). This test is useful for identifying whether significant associations exist between variables.

When a significant association was detected (p-value < 0.05), the Odds Ratio was subsequently employed (Andrade 2023). This measure quantifies the strength of the association, helping to understand the magnitude of the effect and indicating how much more or less likely one group is to experience a particular event compared to another (Hoffmann 2016).

All statistical analyses were performed using RStudio (version 4.3.3; R Foundation for Statistical Computing, Vienna, Austria).

The term “socioeconomic vulnerability”, as defined by the SEMSAs, will be adopted in this manuscript. This definition encompasses broad social factors such as gross household income, family composition, housing conditions, and access to education and healthcare, collected through the household survey conducted by the SEMSAs. For characterization in this study, the following variables were considered: number of household residents, gross family income, respondent’s level of education, housing conditions, and access to both basic and specialized healthcare services.

## Results

### Description of skin phototypes

**Fig. 2:**
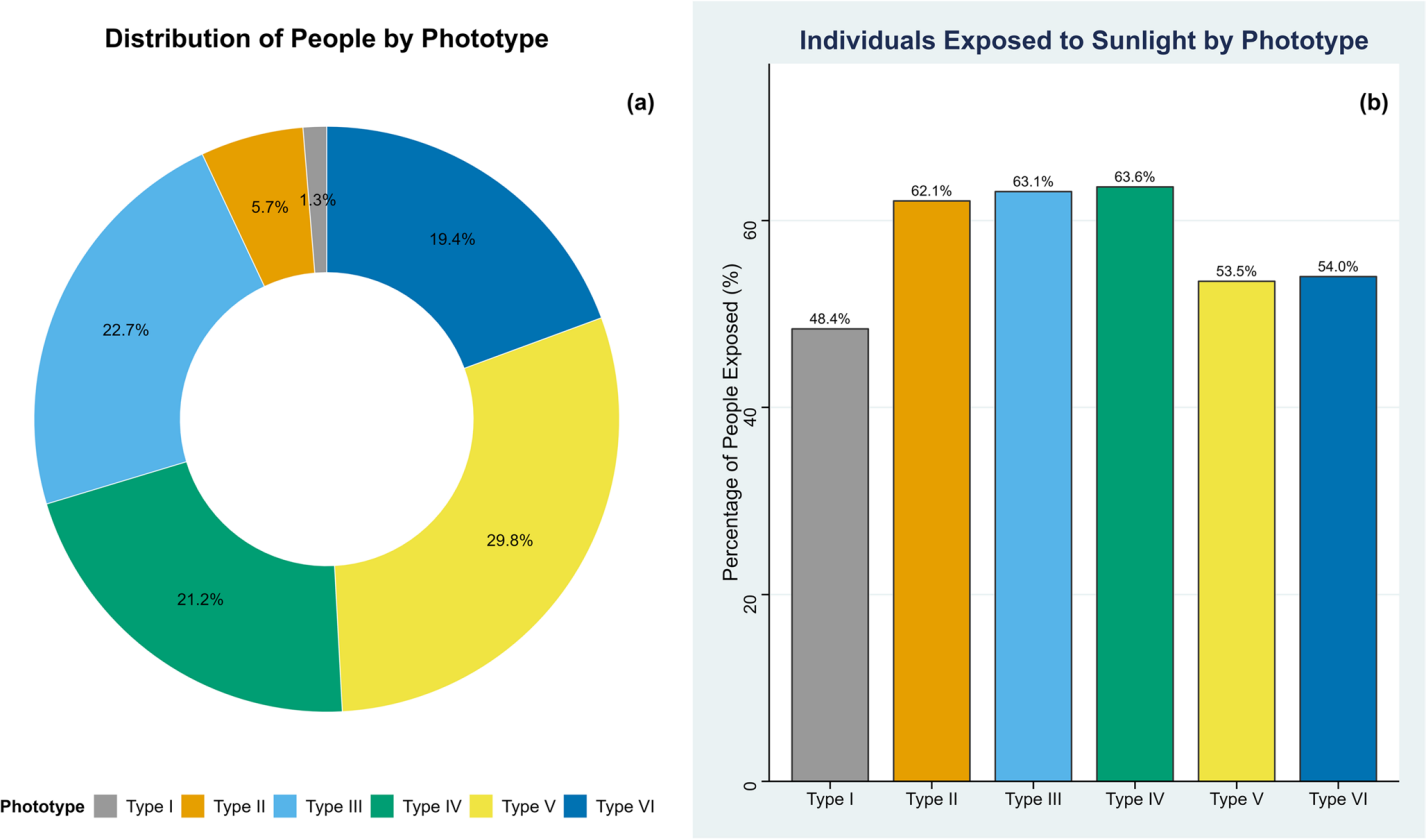
Skin phototype distribution and individuals exposed to the sun between 10:00 AM and 3:00 PM, based on data from the Municipal Health Departments of Manicoré, Apuí, and Novo Aripuanã. (**a**) Percentage distribution of skin phototypes. (**b**) Percentage distribution of individuals exposed to solar radiation by phototype

The phototypes III, IV, and V showed the highest proportions (above 20%) within the sampled population of the Immediate Geographic Region of Manicoré, with phototype V having the highest representativeness at 29.8% (95% CI: 27.5–32.2). Phototypes I, II, and VI represent proportions below 20%, with phototype I having the lowest representativeness at 1.3% (95% CI: 0.9–2.1) (Fig. 2a). Regarding sun exposure between 10:00 AM and 3:00 PM, 48.4% of individuals with phototype I reported having the habit of sun exposure. For phototypes II to IV, this habit is maintained by at least 63% of individuals in each phototype, while for phototypes V and VI, at least 53% also reported being exposed (Fig. 2b).

### Sun exposure habits

**Fig. 3:**
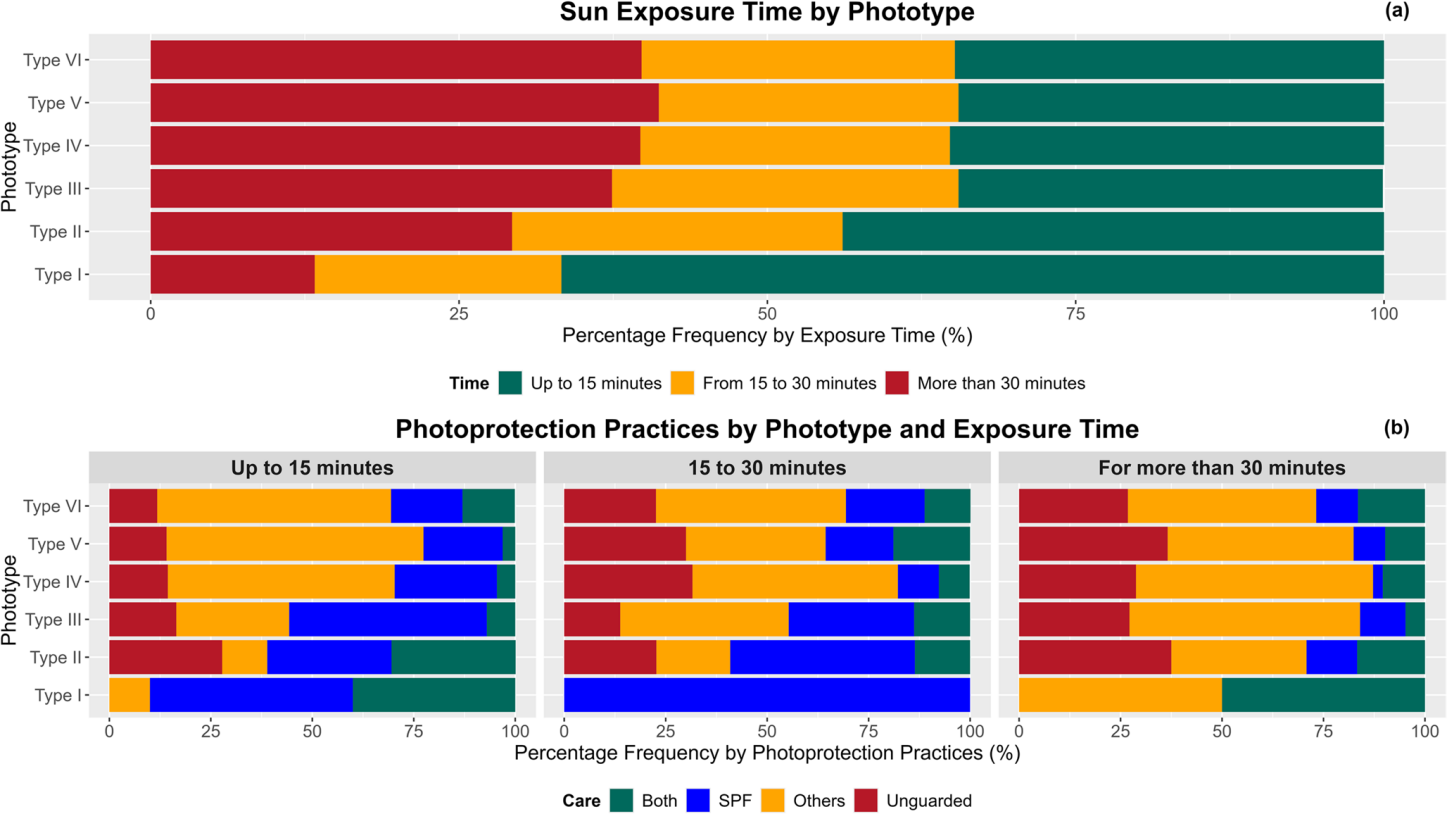
Percentage distribution by phototype of sun exposure duration in (**a**) and photoprotection practices in (**b**), where ‘Both’ indicates the adoption of sunscreen and Others; SPF stands for Sun Protection Factor and ‘Others’ refers to the use of hats, sunglasses, and shirts as photoprotection practices

Figure 3a shows that 67% and 44% of individuals with phototypes I and II, respectively, reported sun exposure for up to 15 min between 10:00 AM and 3:00 PM. For other phototypes, this proportion ranged from 34% to 35%. Exposure lasting 15 to 30 min was similar across all phototypes (20% to 28%), while exposure exceeding 30 min was more frequent among phototypes III to VI, with phototype V standing out at 41%.

Individuals with skin type I never expose themselves to sunlight without protection, regardless of the duration of exposure. For exposures of up to 15 min, 28% of individuals with skin type II do not utilize protection, while a proportion of less than 17% among the other skin types displays the same behavior. In exposures ranging from 15 to 30 min, the highest proportions of unprotected exposure occur among skin types IV (32%) and V (30%). For exposures exceeding 30 min, more than 25% of individuals across each skin type, with the exception of those with skin type I, do not adopt any protective measures. Generally, despite the exposure to solar radiation, at least 72% of individuals in each skin type (excluding skin type I) who are exposed for up to 15 min employ some form of protection, such as the use of sunscreen, clothing, hats, among others. For exposures longer than 30 min, approximately 62% adopt protective practices (Fig. 3b).

### Photoprotection practices and socioeconomic vulnerability

**Fig. 4:**
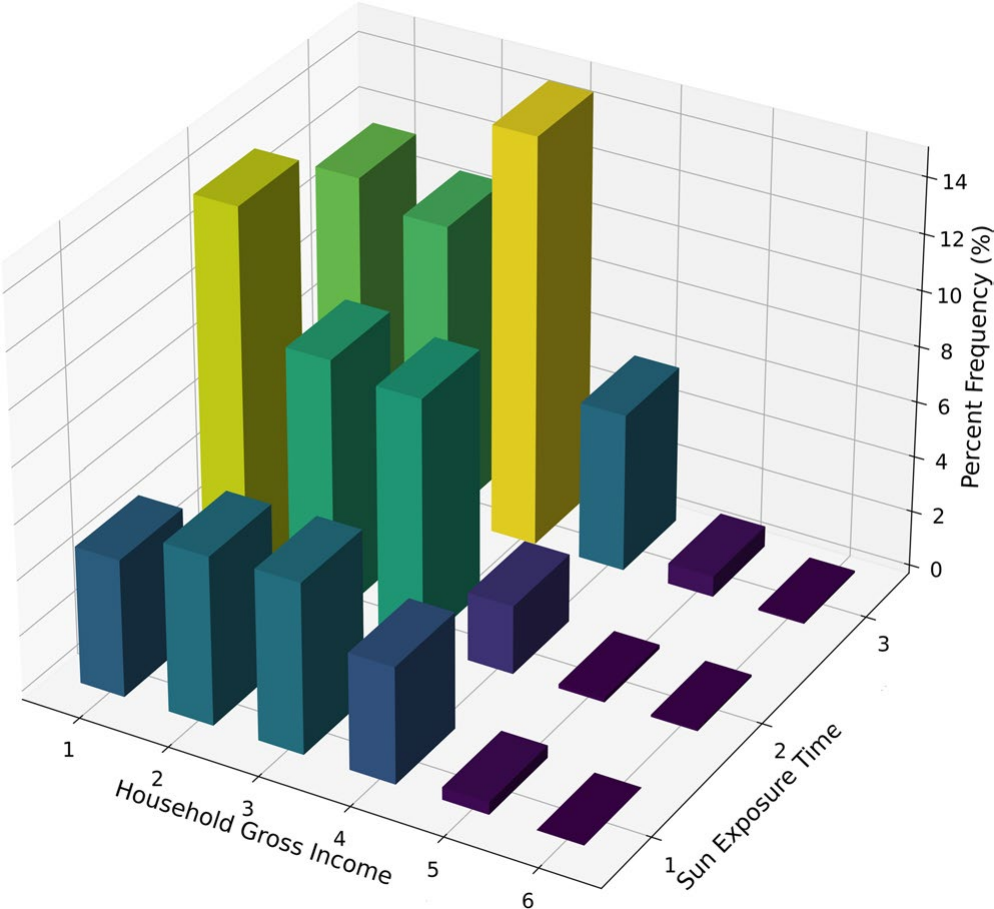
Three-dimensional histogram representing the percentage distribution of the sampled population in terms of gross household income and sun exposure time. On the horizontal plane, the ‘Household Gross Income’ axis ranges from 1 to 6, where the categories represent, respectively, households with incomes below 1 minimum wage (1), equal to 1 minimum wage (2), between 1 and 1.5 minimum wages (3), between 1.5 and 3 minimum wages (4), between 3 and 5 minimum wages (5), and above 5 minimum wages (6). The ‘Sun Exposure Time’ axis defines three intervals: up to 15 min (1), between 15 and 30 min (2), and over 30 min (3). The vertical axis represents the percentage frequency, with colors transitioning from darker tones (blue) indicating lower frequencies to lighter tones (yellow) representing higher frequencies

The initial analysis of sun exposure and household income revealed that just over 50% of the sample is exposed to the sun for more than 15 min and lives on an income below three minimum wages (Fig. 4). However, when considering factors that determine socioeconomic vulnerability, such as access to healthcare, housing conditions, family composition, and education levels, it was found that 94.6% of the sample falls into this category. Although high, this proportion is consistent with the socioeconomic profile of small and medium-sized interior municipalities in the Amazon, where limited healthcare access, precarious housing, low education levels, and insufficient basic sanitation are common. Therefore, this percentage should be interpreted as representative of the study area rather than of the Amazon region as a whole.

**Fig. 5:**
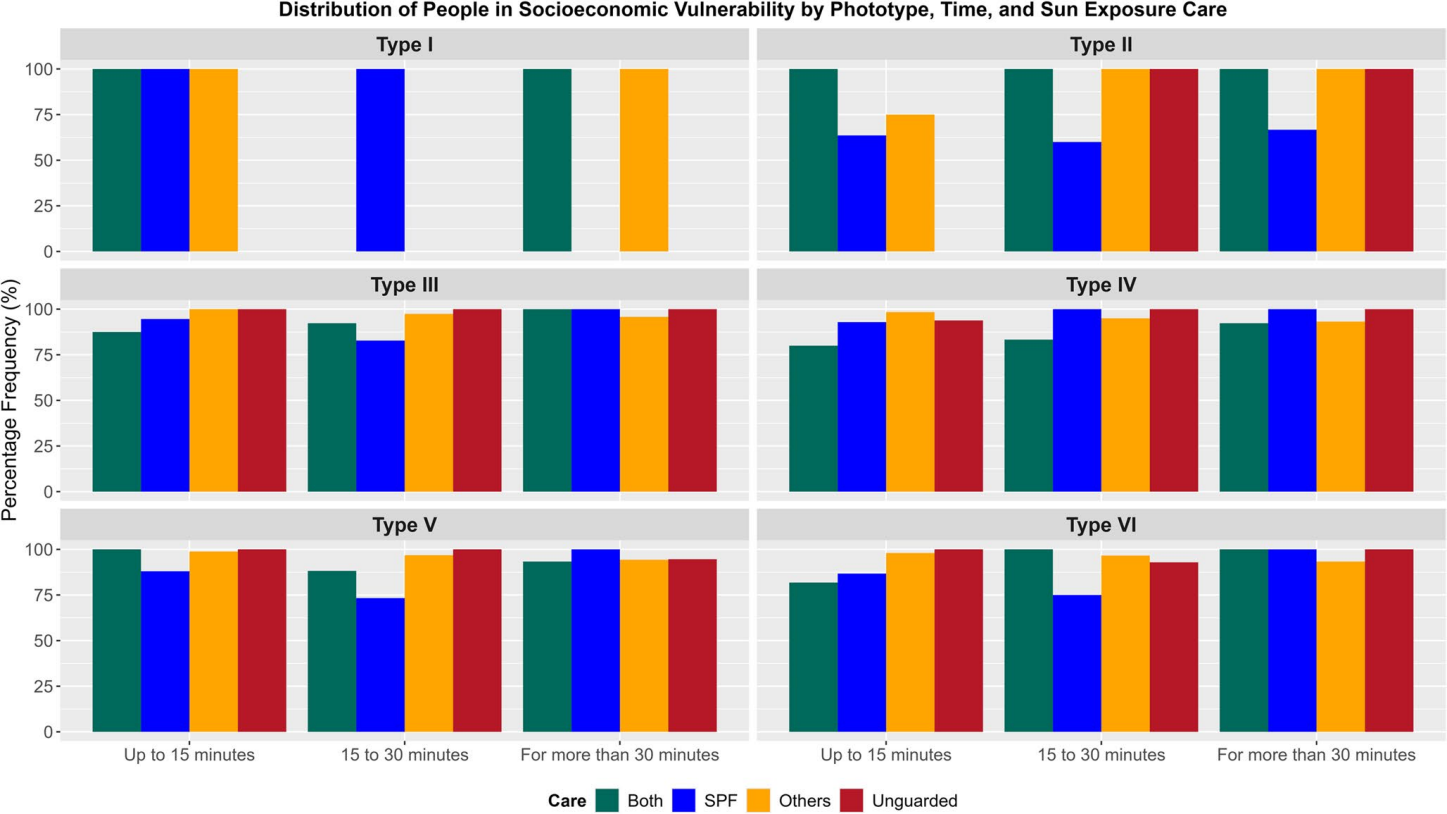
Percentage distribution of individuals in socioeconomic vulnerability and their photoprotection practices. The chart illustrates the relationship between the level of sun protection adopted and socioeconomic conditions, highlighting the behavior of the vulnerable population in terms of sun exposure and the use of preventive measures, such as sunscreen, appropriate clothing, and hats

 Fig. 5 illustrates that individuals with phototype II who exclusively use Sunscreen Protection Factor (SPF) exhibit the lowest percentages of socioeconomic vulnerability, ranging from 60% to 67%, depending on the duration of exposure. For other phototypes, this proportion exceeds 73%, regardless of the duration of exposure.

Individuals who use alternative forms of photoprotection, such as shirts, hats, and sunglasses, are predominantly in a situation of socioeconomic vulnerability, with at least 93%, except for phototype II exposed for up to 15 min, where the percentage is 75% (Fig. 5).

Furthermore, when examining each phototype, Fig. 5 reveals that the minimum percentage of individuals in a socioeconomic vulnerability situation who combine the use of sunscreen (SPF) with alternative photoprotection measures, such as shirts, hats, and sunglasses, is never less than 80%.

Considering the proportions of individuals in conditions of socioeconomic vulnerability who adopt (76%) or do not adopt (24%) photoprotective practices, and of non-vulnerable individuals who adopt (87.8%) or do not adopt (12.2%) such practices, the analyses indicate that the proportion of vulnerable individuals who do not adopt photoprotection measures is significantly higher compared to non-vulnerable individuals. Pearson’s chi-square test with Yates’ continuity correction revealed a statistically significant association between socioeconomic vulnerability and photoprotective practices (*p* = 0.02). The direction of this association indicates that individuals in conditions of socioeconomic vulnerability are more likely not to adopt sun protection measures compared to those who are not in this condition. This result is supported by the obtained Odds Ratio of 2.28, with a 95% confidence interval ranging from 1.12 to 4.63. In other words, socioeconomically vulnerable individuals are 2.28 times more likely not to adopt photoprotective practices compared to their non-vulnerable counterparts. These findings reinforce the premise that socioeconomic vulnerability is a relevant factor underlying lower adherence to photoprotective practices.

## Discussion

### Description of skin phototypes

Individuals with melano-compromised skin (phototypes I and II), characterized by very fair skin tones and low melanin production, are highly susceptible to UV radiation damage. This group represents only 7% of the study sample. Conversely, individuals with melano-competent skin (phototypes III and IV), with fair to medium skin tones and higher melanin production, constitute 43.9% of the sample and exhibit greater protection against UV radiation. Melano-protected individuals (phototypes V and VI), with dark brown to black skin tones, account for nearly 50% of the sampled population and possess the highest level of natural protection against UV radiation, significantly reducing the risk of sunburn. In summary, the higher the melanin concentration (higher phototypes), the greater the natural protection against UV damage (Al-Sadek and Yusuf 2024; Araújo, et al., 2014; Fitzpatrick 1988).

Unlike Fitzpatrick’s classification, which is based on skin phototype and melanin response to UV radiation (Fitzpatrick 1988), the Brazilian Institute of Geography and Statistics (IBGE) categorizes the population by self-declared race/color: white, black, brown, yellow, and Indigenous. In the cities analyzed, the population distribution was 18.1% white, 5.3% black, 72.1% brown, 0.1% yellow, and 4.4% Indigenous (IBGE 2020). While this classification is valuable for demographic purposes, it has limitations for describing physical characteristics and UV sensitivity. In contrast, the Fitzpatrick scale, although not fully capturing the diversity of mixed-race populations in the Amazon, is more appropriate for dermatological and photoprotection research, including the estimation of Minimal Erythemal Dose (MED) (Table 1). It is also recognized by the Brazilian Society of Dermatology (SBD) as a key tool for guiding sun protection policies (SBD 2021b).

**Table 1:** Fitzpatrick scale considering tanning ability and susceptibility to sunburn when exposed to a specific dose of UV solar Radiation. Adapted from Corrêa and Pires (2013)

Phototype	Sunburn	Tanning	MED (mJ·cm^− 2^)
I	Yes	No	20–30
II	Yes	Light	25–35
III	Yes	Medium	30–50
IV	No	Dark	45–60
V	No	Naturally Brown	60–100
VI	No	Naturally Black	100–200

The proposal to include the Fitzpatrick scale in the IBGE census is supported by data highlighting the need to revise the current classification. INCA projects that non-melanoma skin cancer (NMSC) will remain the most prevalent type of cancer in Brazil, with a significant increase of 4.3% points between the 2020–2022 and 2023–2025 triennia (INCA, 2022; INCA, 2019). Although NMSC is less lethal than melanoma, its prevalence is significantly higher (Ferlay, et al., 2021).

The increasing trends in the incidence of non-melanoma skin cancer (NMSC) in Brazil are evident and have consequently resulted in a rise in NMSC-related mortality. According to Nascimento et al. (2022), 27,550 deaths from NMSC were reported in Brazil, with a higher proportion among males (58.1%) and individuals aged 70 years or older, who accounted for 64.3% of recorded cases. Furthermore, the authors highlighted that the Northern and Northeastern regions of the country experienced significant upward trends in mortality rates, reflecting a heterogeneous regional distribution of the disease’s impact across Brazil.

On a global scale, Bray et al. (2024) reported that the estimated incidence of non-melanoma skin cancer (NMSC) in 2022 reached approximately 1.2 million cases worldwide, with around 70,000 deaths attributed to this malignancy. Similarly, Kerob et al. (2024) documented 63,731 NMSC-related deaths globally in 2020, highlighting that this issue has been gaining prominence not only in specific regions but on a global scale. A recent study projected that the incidence, deaths, and disability-adjusted life years (DALYs) associated with NMSC will increase by at least 1.5 times globally between 2020 and 2044 (Hu, et al., 2022).

Regarding malignant melanoma, more than 47,000 deaths were recorded in Brazil between 1979 and 2022, with a significant upward trend of 39 additional deaths per year (Zanoni et al. 2023). In the Northern region, 985 deaths were reported during the same period, also showing a consistent annual increase. In the analyzed cities, only two fatal cases were recorded (INCA 2024; Mélo et al. 2019). Suspected cases of melanoma or non-melanoma skin cancer (NMSC) in the RGIM, except in Humaitá, are referred to specialized centers located in the state capitals of Manaus (Amazonas) and Porto Velho (Rondônia), where mortality statistics likewise indicate rising trends.

The worsening of skin neoplasms in Brazil, particularly in the North, where underreporting is heightened by a lack of specialists and infrastructure (Garnelo et al. 2020), underscores the need to discuss the collection of skin color information (Szklo, et al., 2007) and to reevaluate the scale used in future censuses.

### Factors influencing exposure habits

Differences in sun exposure and photoprotection practices among phototypes stem from melanin levels, which vary across the six phototypes (Al-Sadek and Yusuf 2024; Kullavanijaya & Lim, 2005; Fitzpatrick 1988). Individuals with higher phototypes (III-VI), less prone to sunburn, perceive lower immediate risk, leading to longer sun exposure (Rigel, et al., 2022; Young et al. 2017).

The high proportions of prolonged sun exposure in the interior cities of Amazonas (Kütting and Drexler 2010; Radespiel-Tröger, et al., 2009; Marehbian, et al., 2007) are linked to predominant occupational activities such as subsistence farming, fishing, livestock rearing, natural resource extraction, handicrafts, and services, all tied to local resources and traditional lifestyles (Costa, et al., 2022; Ceballos et al. 2014; Lima and Pozzobon 2005). The frequent use of motorcycles and bicycles as urban transportation also increases sun exposure during daily commutes. These factors, reflecting the region’s socioeconomic and cultural conditions, emphasize the need for effective photoprotection practices when assessing sun exposure behaviors across different phototypes in the population (Modenese et al. 2018). Our findings corroborate this relationship, as we observed high proportions of individuals exposed for more than 30 min, particularly among phototypes III to VI, along with low adherence to regular sunscreen use. These results directly reflect the role of occupational and cultural activities in shaping the sun exposure patterns identified in our sample.

Regarding the burden of NMSC attributable to occupational exposure to solar ultraviolet radiation, the World Health Organization (WHO) estimated that, in 2019, approximately 18,960 out of 65,440 NMSC deaths were linked to such exposure (Pega et al. 2023; Sung et al. 2021). Pega et al. (2023) highlighted that between 2000 and 2019, NMSC deaths due to occupational exposure to solar ultraviolet radiation increased by 87.9% globally. According to the authors, males carried a larger burden, and the number and rate of deaths increased with age, peaking at 65–69 years, due to disease latency.

It is important to note that part of the increase in non-melanoma skin cancer incidence may be related to demographic changes, particularly population aging. This aspect is especially relevant, as most cases tend to manifest in older age, given that skin cancer—whether melanoma or non-melanoma—is strongly associated with the cumulative lifetime dose of UV radiation. However, international studies indicate that even after age-standardization, incidence rates of non-melanoma skin cancer continue to rise, suggesting that additional factors, such as increased UV exposure and improved diagnostic capacity, also play a significant role in this trend (Bray et al. 2024; Hu et al. 2022; Pega et al. 2023).

These global data underscore the significant impact of both occupational exposure and demographic aging on the increase in NMSC deaths. The reality of the Amazon region, characterized by economic activities dependent on long outdoor work hours, exacerbates the susceptibility of local populations. The lack of effective preventive measures and specialized healthcare infrastructure, such as the scarcity of dermatologists, further worsens the situation. Therefore, it is urgent to implement photoprotection strategies tailored to regional conditions and to promote awareness campaigns focused on prevention, especially among exposed workers, to mitigate the impacts of solar exposure and reduce the future burden of NMSC in the Amazon and other regions with similar characteristics.

The provision and retention of healthcare professionals in primary care within hard-to-reach areas of Amazonas remain a persistent challenge. Since the 1970 s, public policies have sought to address the difficulty of attracting and retaining general practitioners and, more notably, specialists in the Amazon region through programs aimed at both training and retaining these professionals (Dolzane and Schweickardt 2020). This issue negatively impacts municipalities in the state of Amazonas due to factors such as limited access to communities, population dispersion, vast geographical distances, inadequate local infrastructure, and the lack of specialized training for professionals working in this context (Garnelo et al. 2020).

The establishment of the *Programa Mais Médicos* (More Doctors Program) by the Brazilian federal government in 2013 has brought significant changes to this scenario. The program’s primary goal is to expand access to primary healthcare in priority areas of the Unified Health System (SUS), particularly in remote, vulnerable, or underserved regions, and it has facilitated notable progress in addressing these longstanding challenges (Lima, et al., 2016).

In this context of progress, countries such as Australia, the United Kingdom, and Canada have implemented innovative strategies by integrating general practitioners into the care and prevention of skin neoplasms. Training these professionals to identify suspicious lesions at early stages has proven effective, with studies showing that routine skin examinations are associated with a 59% reduction in melanoma-specific mortality and a 36% reduction in all-cause mortality compared to patient-detected cases (Green et al., 2021). Furthermore, research conducted in rural Australia indicates that general practitioners with greater clinical experience achieve higher diagnostic accuracy in detecting skin cancer (Dopheide et al. 2025). These findings help explain the more favorable outcomes observed in skin cancer control in these countries. Despite these successful international experiences, a central challenge in Brazil remains the adoption of individual photoprotection measures, particularly the use of sunscreen.

Proper application, including the selection of appropriate formulations for each phototype and adequate dosage (Passeron et al. 2021; Krutmann et al. 2020), is essential for protection against UVB-induced sunburn, photoaging, skin cancer, DNA damage, and UVA-aggravated photodermatoses (Shetty et al. 2023; Krutmann et al. 2021; Passeron et al. 2021; Eleftheratos et al. 2020; Saucedo et al. 2020; Moyal and Fourtanier 2001). Despite its relevance throughout the year, studies show decreased sunscreen use during winter, when the need for protection is often underestimated (Janssen et al. 2012; Benvenuto-Andrade et al. 2005). In our study population, predominantly composed of melano-competent and melano-protected individuals, natural melanin levels confer some protection against UVB; however, these groups remain susceptible to UVA-induced hyperpigmentation and visible light-related pigmentation (Dlova et al. 2019; Kohli et al. 2018; Duteil et al. 2014; Mahmoud et al. 2010). The low adherence to sunscreen use observed in our sample highlights the need for targeted educational strategies not only for more sensitive phototypes but also for higher phototypes, which have historically been less informed about photoprotection (Passeron et al. 2021; Marionnet et al. 2017; Suarez et al. 2017). Beyond avoiding sun exposure at peak hours, appropriate sunscreens with UVA-PF/SPF ratios ≥ 2/3 and tinted formulations for visible light protection are essential to prevent hyperpigmentary disorders and other skin damage (Seck et al. 2023). Awareness campaigns adapted to regional characteristics are crucial, aligning global scientific evidence with local realities—particularly in regions such as the southern mesoregion of Amazonas, where UVA irradiance has shown significant upward trends (Alves et al. 2025).

In Brazil, public policies for sunscreen use remain limited. Law 14,539/23, effective since September 2023, established the National Campaign for the Prevention of Inappropriate Sun Exposure, aiming to raise awareness about the risks of sun exposure and ensure access to sunscreen through the SUS for individuals with conditions aggravated by solar radiation and outdoor workers (Brasil 2023). Meanwhile, campaigns such as “Dezembro Laranja,” promoted by the Brazilian Society of Dermatology (SBD), and initiatives from INCA highlight the importance of photoprotection (Santos, et al., 2023; SBD 2021b; SBD 2021a).

Bill 8033/17, approved by committees in the Chamber of Deputies, can also be highlighted. It proposes providing free sunscreen and sunglasses through the SUS for individuals with albinism (Brasil 2017). T. The bill also includes the provision of clothing with sun protection factor (SPF 50 or higher) for this population, emphasizing the need for special care due to their heightened susceptibility to UV radiation-induced damage.

In Amazonas, the “Atualiza Pescador Program” distributed 1,100 sun protection kits to fishers in 19 municipalities in 2023. In 2024, the program was expanded, with 2,100 kits distributed. Future phases aim to reach all 62 municipalities in the state. The kits include UV-protective shirts, sunscreen, and straw hats, safeguarding the health of fishers exposed to the sun during their daily activities (SEPROR 2023).

Although photoprotection campaigns are uncommon in the interior cities of Amazonas, particularly within the RGIM, the intense and frequent sun exposure in the region demands greater attention from health authorities (Nascimento, et al., 2022). To expand these initiatives, it is essential to continue investing in public health programs and reducing economic barriers to accessing sunscreen, especially for low-income populations and outdoor workers (Costa, et al., 2022).

### Photoprotection practices in contexts of socioeconomic vulnerability

This high percentage of individuals in conditions of socioeconomic vulnerability, although striking, is supported by data and studies on the Amazonian reality. Several investigations indicate that, despite the economic advances observed throughout the twentieth century, the region remains marked by high poverty rates, per capita income below the national average, deficiencies in basic sanitation, inequalities in access to healthcare and education services, and precarious housing conditions. Recent data show that approximately 20.9% of the Amazonian population lived below the poverty line in 2019, while about 40% lacked access to safe drinking water and nearly 80% did not have access to sanitation services. These indicators confirm that very high levels of socioeconomic vulnerability are consistent with the reality experienced in small and medium-sized municipalities in the interior of the Amazon. Therefore, the result found in this study should not be interpreted as an exception but rather as a reflection of the structural inequalities that have historically affected the region (Rodrigues and Silva 2023).Prolonged sun exposure in the RGIM, often associated with occupational activities and unfavorable economic conditions (Modenese et al. 2018), demands attention from local authorities due to its role in increasing the risk of skin cancer, including melanoma and non-melanoma types (Mesquita, et al., 2020). Even in less severe scenarios, less harmful skin lesions may still occur (Saucedo et al. 2020). Investing in campaigns and subsidies to promote adherence to photoprotection practices emerges as an effective strategy to prevent these pathologies (Mesquita, et al., 2020).

Early diagnosis of skin neoplasms and immediate treatment initiation are crucial for a favorable prognosis (Passeron, et al., 2021). However, this poses a significant burden on public resources, as low-income populations, who predominantly rely on the SUS, face slower diagnostic processes (Mesquita, et al., 2020). These processes include outpatient consultation, referral, biopsy, histopathological analysis, diagnosis, and treatment, where delays at any stage can be life-threatening. In contrast, individuals with higher socioeconomic status have greater access to superior treatments and more effective photoprotection measures (Abdel-Rahman 2020; Mesquita, et al., 2020).

The treatment of skin cancer, the most common type in Brazil, places a significant financial burden on the SUS. In the state of São Paulo, the average annual cost per patient was estimated at R$ 1,172 for non-melanoma skin cancer and R$ 13,062 for cutaneous melanoma in the SUS, while in private healthcare systems, the costs were R$ 1,040 and R$ 26,668, respectively (Souza, et al., 2011). Despite lower individual costs, the high prevalence of non-melanoma skin cancer imposes a significant financial burden on the healthcare system. This impact is further exacerbated by rising medical costs and the complexity of resources required for melanoma cases. The treatment costs cited above refer to the state of São Paulo (Souza et al. 2011), which has the largest healthcare infrastructure in Brazil. Although these values cannot be directly extrapolated to Amazonas, the economic burden is likely comparable—or even greater—when considering additional challenges in the region, such as patient referrals to Manaus or Porto Velho, the scarcity of specialists, and the higher logistical costs of care in remote areas. These factors suggest that the treatment of skin cancer represents a significant economic challenge for the Amazonian health system as well.

Recent advancements, such as the approval of photodynamic therapy by the SUS, offer new perspectives. This innovative treatment, developed by the University of São Paulo (USP), represents a significant shift in the approach to skin cancer (Salvio, et al., 2022). This technique enables the treatment of non-melanoma skin cancer without the need for surgery, using light to activate a photosensitizing drug that selectively destroys cancer cells (Salvio, et al., 2024; Salvio, et al., 2024). In addition to being less invasive, it reduces post-treatment complications and may generate cost savings for the SUS.

This scenario highlights the importance of preventive strategies and early diagnosis, which are more economically viable and reduce the public health impact of skin cancer. Educational campaigns, photoprotection policies, and equitable access to innovations such as photodynamic therapy in the SUS underscore the role of scientific advancements in promoting more accessible and effective cancer treatment.

However, the adoption of these preventive strategies is not uniform across the population. The Odds Ratio of 2.28, with a 95% confidence interval ranging from 1.12 to 4.63, indicates that individuals in socioeconomic vulnerability are 2.28 times more likely not to adhere to photoprotection practices compared to those in more favorable conditions. This underscores the relevance of socioeconomic vulnerability as a determining factor for lower adherence to these practices (Oliveira 2013).

Socially vulnerable individuals diagnosed with melanoma are at higher risk of not receiving surgical treatment and of presenting the disease at an advanced stage (McCampbell, et al., 2024). This scenario reflects the barriers to accessing healthcare services and specialists faced by these populations, as documented in the literature (Abdel-Rahman 2020; Garnelo et al. 2020).

Although NMSC has a relatively lower impact in terms of mortality rates and numbers, given its favorable prognosis and low metastasis rate, its incidence has shown consistent growth, both in regional contexts, such as those analyzed in this study, and on an international scale (Ciuciulete et al. 2022). Globally, NMSC is now responsible for a greater number of deaths than melanoma, widely recognized as the most aggressive form of skin cancer (Arnold, et al., 2022; Bray, et al., 2024).

Additionally, experts highlight that NMSC cases are significantly underreported, suggesting that the true burden of this neoplasm may be even greater than current estimates indicate (WCRFI 2022). This particularity is especially relevant in the context of this study, considering the previously mentioned specificities of the analyzed region.

Skin cancers represent a significant public health issue in medical practice, despite being largely preventable and treatable. However, preventing the progression of these neoplasms requires a collective effort. The reasons for recent reductions in incidence remain under debate but likely reflect a combination of lifestyle and social behavior changes, driven by educational campaigns that encourage and raise awareness about the importance of adopting effective photoprotection practices.

These practices include avoiding sun exposure during peak intensity hours, seeking shade, wearing sun-protective clothing and wide-brimmed hats, and applying sunscreen correctly and regularly. Raising public awareness about these measures is essential to reducing the burden associated with these diseases.

## Conclusions

The findings of this study emphasize the urgent need for targeted public health policies to mitigate sun exposure risks in the Immediate Geographic Region of Manicoré. The predominance of melano-protected and melano-competent phototypes, combined with low sunscreen adherence and socioeconomic vulnerabilities, underscores the importance of overcoming economic barriers through educational campaigns and the distribution of photoprotection products. Incorporating the Fitzpatrick scale into national surveys could improve the identification of at-risk groups, supporting more effective interventions. Finally, expanding access to dermatological care—through mobile clinics or the training of non-specialists in remote areas—represents a feasible strategy to reduce underreporting and improve early detection and management of skin cancer.

## Data Availability

The datasets generated and/or analyzed during the present study are not publicly available as they pertain to a local database, and the corresponding author does not have permission to share them.

## Code availability

Not applicable for that section.
